# Event and Comment

**Published:** 1918-05

**Authors:** 


### DENTAL REGISTER

Vol. LXXII MAY, 1918 No. 5

### EVENT AND COMMENT

**How Can We Meet the Demand of Dentistry?**

Owing to the heavy demands of the war situation we have already lost to active practice for the civilian people over five thousand dentists, which is about one-ninth of all the active practitioners of the United States and its island possessions. It is also probable that most of the graduates for this year will be conscripted for army service.

The Preparedness League is asking that the profession hold itself ready to put the teeth of all the soldiers enlisting this year in good order before they are sent over seas. While it is true that a considerable part of this work will be done by enlisted dentists, there will still be a big job for the profession if it meets the expectations of the Preparedness League, which is asking that one million operations be contributed by the profession. This means that an average of thirty operations must be made by every dentist in the country. Some will meet this and more, perhaps, but to get all to comply will be almost impracticable.

In view of the well known fact that we are woefully short of dentists to meet the ever increasing demand for the various lines of conservation dentistry, it would seem that some practicable means of preventive dentistry must be discovered very shortly or we are destined to fail to meet the expectations of those who are being educated more generally today than ever before to appreciate the true value of their teeth.

Preventive dentistry today has no serious place in the practice of most dentists, as the great majority are busily occupied in doing the necessary rescue and repair work, which is so insistent in its demands that it obscures the vision of the idealists in our profession and consequently little progressive effort is made to put to the test the conceptions we have already developed of a day when dental disease shall not be the most universally prevalent of all diseases.

It then becomes necessary that we adopt very soon some method of increasing the power or capacity of the profession to care for the ever increasing demand for practical dentistry. Can we hope that present methods of treatment and repair can be made simpler and in this way require less time, so that each dentist may care for a larger number of patients? Anyone who reads our periodicals or attends our dental society meetings will soon realize that the ideal that is being everywhere emphasized is for more careful and more exact technical handling of all restorative procedures. Our young men are being trained in college to make this their main ambition. Rapid dentistry is everywhere discredited. We are losing out in the quick sort of operations and are straining every nerve to do the better forms, which obviously consume time, rather than enlarge one's capacity for an increased output. Another discouraging outlook is the fact that owing to present war conditions, and the advancement in educational standards, fewer rather than more young men are seeking dental qualifications, and there is going to be a great lack of dental service unless some measures are adopted very soon to take care of a situation that is becoming more serious every day.

It has been thought that the education of a class of women, dental hygienists, who, working in association with dental practitioners as specialists in prophylaxis of the teeth alone, would not only lighten the work of the dentist, but also prevent, to a degree, the increased need of operative and therapeutic treatment. The fact that only five states have so far legalized such practitioners, and that only three institutions have so far announced courses of training for the dental hygienist, and comparatively few women have applied for this training, does not promise sufficient relief to compensate at all for the losses occasioned by the war and other causes above enumerated.

We do not wish to be considered pessimistic in regard to this situation, but we believe it is a matter that needs to be recognized; and some proper and adequate measures should be put under way at an early date that shall prevent us from a situation that is acutely troubling some of the countries of Europe today and will probably affect us more seriously than we now think. We sincerely hope it will not come in such a form as to affect our standards of technical skill which is the most natural and usual form of solution. If we are required to do a big business with inadequate help we shall surely degenerate in the quality of the service we can or do render.

One solution that may help at least to tide us over the present emergency may be found in a better organization of the business conduct of the dental office. At the present time the average dentist thinks it economical to do himself as much of the work required in his office as possible, even to the extent of janitor service, or dispensing with the usual office maid or assistant. Is it not practicable to employ a more skillful grade of office assistants even at increased cost? An intelligent and tactful woman can be taught to do many things in a dental practice that consume the time of the dentist; even to the extent of doing much of the laboratory technic work. If so, this would relieve the dentist of care and give him the much needed time for more chair work. If the practice would justify the addition of a dental hygienist, she would help to keep up the standard of efficiency and inaugurate preventive dentistry. Then, too, a laboratory technician who could take over all the mechanical technic work and relieve the operator in this department of all care and responsibility would be a material resource in efficiency. If our offices could be organized and conducted on this basis it would be possible to increase our output of service by a half without the addition of more qualified dentists than we now have; and good business management along this line would tend to better standards, as well as take care of what seems like a very difficult problem. Let's study this problem.

